# Draft Genome Sequence of a Terrestrial Planctomycete, *Singulisphaera* sp. Strain GP187, Isolated from Forest Soil

**DOI:** 10.1128/MRA.00956-20

**Published:** 2020-12-10

**Authors:** Maureen A. Morrow, Grace Pold, Kristen M. DeAngelis

**Affiliations:** a Department of Biology, State University of New York at New Paltz, New Paltz, New York, USA; b Department of Natural Resources Management and Environmental Sciences, California Polytechnic State University, San Luis Obispo, California, USA; c Department of Microbiology, University of Massachusetts, Amherst, Massachusetts, USA; University of Maryland School of Medicine

## Abstract

Here, we present the draft genome sequence of a novel species of the genus *Singulisphaera* (phylum *Planctomycetes*, family *Isosphaeraceae*) isolated from soil. *Singulisphaera* sp. strain GP187 has a relatively large mobilome and numerous novel genes that may contribute to the production of bioactive molecules.

## ANNOUNCEMENT

Culture-independent analysis reveals that *Planctomycetes* is the fifth most abundant bacterial phylum in global soil samples (1), yet this phylum remains underrepresented in axenic cultures, and a large majority of these cultures are derived from aqueous environments (2). Aqueous planctomycetes are hypothesized to have evolved from terrestrial species (3).

*Singulisphaera* sp. strain GP187 was isolated on 3 June 2014 from the Harvard Forest, a temperate forest ecosystem in Petersham, MA (42.54°N, −72.18°W). Organic horizon soil was pretreated with 6% yeast extract plus 0.05% SDS (4), plated onto oatmeal medium, and incubated aerobically at 20°C, with colonies appearing after 8 days. GP187 was the only *Planctomycetes* strain of the hundreds of isolates from this site (5) and thus was subjected to whole-genome sequencing.

GP187 was grown aerobically on Reasoner’s 2A (R2A) medium (pH 7) (6). Genomic DNA was purified using a modified cetyltrimethylammonium bromide (CTAB) procedure (7) but was not sheared or size selected. The draft genome sequence was generated at the DOE Joint Genome Institute (JGI). A PacBio SMRTbell library was constructed and sequenced on the PacBio RS platform, generating 407,937 reads (*N*_50_, 3.6 kbp). The filtered raw reads (675.3 Mbp) were assembled using HGAP v2.3 _p5 (protocol version, 2.3.0; method, RS HGAP Assembly.3, smrtpipe.py v1.87.139483) (8). The final draft assembly contained 5 contigs in 5 scaffolds (*N*_50_, 6.278 Mbp), estimated as 99.61% complete and 5.81% contaminated using CheckM v1.0.18 (9) in KBase (10). The input read sequencing depth was 72.8×. Gene annotations were completed within the JGI’s Integrated Microbial Genomes (IMG) platform (11) and KBase. Default parameters were used for all software.

The genome is 10,689,158 bp (G+C content, 63.07%) and is predicted to encode 8,388 proteins (36.6% without predicted function), 8 rRNA operons, and 110 RNA genes (24 rRNAs, 64 tRNAs). GP187 has the largest genome of cultured *Isosphaeraceae* strains and the second largest genome of cultured *Planctomycetes* strains (12).

Phylogenetically, the closest species to GP187 is the aquatic Singulisphaera acidiphila DSM 18658 (13). These strains share 98.84% homology for 16S rRNA genes (average as determined by searching public RNA isolates with IMG BLAST) and 86.7% whole-genome average nucleotide identity (ANI) (IMG pairwise ANI). *Isosphaeraceae* genome sequences characteristically carry large plasmids; GP187 harbors a putative plasmid of 63.8 kb (G+C content, 61.8%; a lower G+C content is typical of plasmids [14]). The subtilisin gene open reading frame spans, without gaps, the ends of contig5 and shares 82% homology with the plasmid-encoded subtilisin gene of *S. acidiphila* DSM 18658, suggesting that contig5 is a circular plasmid.

GP187 contains 60% more mobilome-associated genes (186 [2.2% of protein-encoding genes] versus 117 [1.5%], respectively) and 78% more genomic islands (41 versus 23, respectively) than *S. acidiphila* DSM 18658, as predicted by IslandViewer4 (15). GP187 has a greater potential to synthesize specialized metabolites, given that novel genes of this species are categorized as giant genes (≤5,000 bp with Kbase RAST annotation) (16) or are located in biosynthesis gene clusters (17) more often than in *S. acidiphila* DSM 18658 (Table 1).

**TABLE 1:** Differences in select characteristics between *S. acidiphila* DSM 18658 and *S. acidiphila* sp. GP187

Strain	Genome size (Mbp)	No. (%) of:			
		Protein coding genes^*a*^	Genes without predicted function^*a*^	Giant genes without predicted function^*b*^	Genes without predicted function in BGCs^*c*^
*S. acidiphila* DSM 18658	9.76	7,576 (98.6)	2,578 (33.6)	7 (20.6)	76 (29.8)
*S. acidiphila* sp. GP187	10.69	8,388 (98.7)	3,110 (36.6)	14 (35.0)	104 (36.4)

a Percentage of total protein coding genes (IMG).

b Percentage of giant genes (KBase).

c Percentage of total genes in biosynthetic gene clusters (BCGs) (IMG).

This genome sequence will contribute to our understanding of terrestrial species of *Planctomycetes*, a phylum abundant in soil but underrepresented in isolate genome analysis. Analysis of this genome sequence may elucidate its ecological role in terrestrial ecosystems, identify evolutionary relationships between terrestrial and aquatic *Singulisphaera* species, and contribute to the discovery of novel secondary metabolites.

### Data availability.

This whole-genome sequence was deposited at DDBJ/EMBL/GenBank under the accession number NZ_FSRB00000000.1. The raw data were deposited in the JGI GOLD under the project number Gp0151081 and in the Sequence Read Archive under the accession number SRX2158412. The JGI annotation is found at https://img.jgi.doe.gov/cgi-bin/m/main.cgi?section=TaxonDetail&page=taxonDetail&taxon_oid=2695420965#.
